# Exploring the Unmet Needs of Cancer Caregivers in India: A Cross-Sectional Survey

**DOI:** 10.7759/cureus.62159

**Published:** 2024-06-11

**Authors:** Shrikanth Muralidharan, Farha R Sikalgar, Deepak B, Monali R Nikalje, Tulsi Subramaniam, Manasvi Kumar

**Affiliations:** 1 Research, National Institute of Naturopathy, Pune, IND; 2 Community Medicine, ZVM Unani Medical College and Hospital, Pune, IND; 3 Clinical Naturopathy, National Registration Board, Ministry of Ayush, Pune, IND; 4 Dentistry, Symbiosis Medical College for Women and Symbiosis University Hospital and Research Centre, Pune, IND; 5 Health Care, School for Skill Development and Allied Health Sciences, Bharati Vidyapeeth (Deemed to be) University, Pune, IND

**Keywords:** india, consequences, catcon questionnaire, head and neck cancer, cancer caregiving experiences

## Abstract

Introduction

Caregivers of individuals with head, neck, and face cancer play a pivotal role in providing support, yet they face distinct challenges. This study aims to explore caregiving experiences and consequences among this population.

Methods

A multicentric cross-sectional study involving 200 caregivers using a convenience sampling method was conducted in Pune. Caregivers involved in patient care were included in head, neck, and face cancer. The Cancer Caregiving Consequences Inventory (CaTCoN) questionnaire was utilized to assess various dimensions of caregiving. Ethical clearance was obtained from institutional review boards.

Results

The demographic profile revealed that 89% of caregivers were spouses/partners, primarily females (77%), aged 25-40 (81.5%), and urban dwellers (68.5%). Caregivers were predominantly married or cohabiting (100%) and had children (95%). Most had a graduate-level education (97.5%) and were employed full-time (82.5%). The mean scores from the CaTCoN questionnaire highlighted substantial caregiving responsibilities, with significant associations found in multivariate regression analysis. Spouses/partners showed stronger correlations with increased workload, personal growth challenges, financial difficulties, and the need to maintain an everyday life.

Conclusion

This study comprehensively explains caregiving consequences among head, neck, and face cancer cases. The predominance of spouses/partners as caregivers emphasizes the need for targeted interventions to address their unique challenges. The study highlights the demanding nature of caregiving, with potential positive outcomes.

## Introduction

Caregivers play a vital role in supporting patients with advanced cancer, but they too face challenges stemming from the patient's illness, often with unmet supportive care needs. A recent review highlighted prevalent needs among informal caregivers, focusing on information needs covering illness, treatment, and care-related information [1]. These needs fall into social, cognitive, and psychological categories [2]. The total number of needs appears consistent over time, while specific needs evolve, the management of stricter aspects of the patient's behavior increases, and the "need for knowledge about the disease" decreases over time [3]. A high number of unmet needs correlate with psychological distress and overall caregiver burden [4]. Factors like lack of training, low support availability, discrepancies between caregiver and patient reports, and challenges in managing medical and non-medical care activities contribute to increased burden [5]. Utilization of support services is infrequently explored, but studies show approximately one-third of utilization, with factors like depressive symptoms, anxiety, preparedness, minority status, shorter caregiving duration, and higher stress burden influencing interest in such services [6]. Surprisingly, informal sources like family and friends are more commonly used by caregivers, with professional sources being the least utilized and perceived as less helpful [7]. Understanding and addressing these complexities are essential to improving the holistic support for family caregivers in the challenging landscape of advanced cancer care. Hence, the present cross-sectional study was carried out to explore the caregiving consequences among head, neck, and face cancer cases in Indian sets. The study aimed to understand the different aspects and impacts of caregiving for head, neck, and face cancer patients. It focused on practical tasks, emotional well-being, and the overall effect on caregivers' lives.

## Materials and methods

The research inquiry aimed to elucidate the intricacies of challenges encountered by caregivers within the context of palliative care for patients afflicted with head, neck, and face cancers. Employing a cross-sectional design from August 2023 to December 2023, the study anticipated a sample size of 384 caregivers, assuming a 50% prevalence rate, to ensure robust statistical analysis. However, data from only 200 caregivers were acquired due to non-participation and incomplete questionnaire submissions. Ethical clearance was meticulously obtained from the Ethics Committee Maharashtra Education Society, Azam Campus (letter 38/EC2023) before the commencement of the study, underscoring the commitment to ethical research conduct. The inclusion criteria for participants in the study encompassed caregiving adults actively providing care for individuals diagnosed and treated for head, neck, and face cancers within the preceding year, irrespective of their relationship to the patient. Only caregivers aged 18 years or older were considered eligible for inclusion. The study was conducted at a single palliative care center in Pune. Thus, participants had to reside within the city limits to facilitate ease of access for data collection. Conversely, exclusion criteria involved caregivers unwilling to participate or provide informed consent for the study. Additionally, caregivers who did not possess the capacity to comprehend and respond to the questionnaire due to language barriers, cognitive impairments, or any other reasons were excluded from participation. Caregivers who were currently undergoing treatment for any mental health conditions or experiencing acute distress were also excluded, to ensure the validity and reliability of the collected data. The Cancer Caregiving Consequences Inventory (CaTCoN) questionnaire was used in the study [8]. It was translated into the local language before administration. Before data collection, written informed consent was obtained from all study participants. The data was coded and entered into Microsoft Excel 2019 (Microsoft Corporation, Redmond, USA) for organization and integrity. SPSS 24.0 (IBM Corp, Armonk, NY, US) was utilized for statistical analysis, primarily employing multivariate techniques to examine the relationship between demographic variables and caregiving challenges reported by respondents. All statistical tests were performed with a predetermined significance level set at p < 0.05 to ascertain the statistical significance of the findings.

## Results

In the present study, most caregivers were spouses (89%) and predominantly female (77%), with 81.5% aged between 25-40 years. All caregivers were married or cohabiting, and 95% had children. Most resided in urban areas (68.5%) and had a graduate-level education (97.5%). Professionally, 58.5% were salaried, while 82.5% were employed full-time (Table 1).

**Table 1:** Demographic characteristics of the caregivers

Characteristics	N	%
Caregiver		
Primary	200	100
Relationship with the patient		
Partner (wife/husband)	178	89
Children	22	11
Gender		
Female	154	77
Male	46	23
Age (years)		
25-40	163	81.5
41-50	37	18.5
Marital status		
Married/cohabiting	200	100
Have children		
No	10	5
Yes	190	95
Place of living		
Urban	137	68.5
Rural	63	31.5
Level of education		
Primary schooling (<1 year)	5	2.5
Graduate (1-3 years)	195	97.5
Profession		
Self-employed	40	20
Salaried	117	58.5
Un-skilled worker	77	38.5
Employment		
Full time	165	82.5
Part-time	35	17.5

The CaTCoN questionnaire outcomes showed that 68% of caregivers provided much practical assistance, with a mean score of 71.9±21.34. Psychological support was another significant area, with 42% providing a lot, resulting in the highest mean score of 78.8±23.4. In contrast, 49% provided no personal care. Caregivers reported high responsibility for home care (80%), frequent transportation for therapy (87%), and significant stress (73%) due to caregiving, with mean scores reflecting substantial stress and impact on physical health (63.4±12.34 and 22.5±14.28, respectively). Financial difficulties were prevalent, with 90% bearing adverse economic situations and 84% needing financial counseling. Only 10% required psychological support. The need to maintain a "normal" life was significant, with 60% expressing a high degree of this need, but only 10% felt they could achieve it to a high degree. Multivariate regression analysis highlighted that female caregivers and spouses faced greater workloads, personal growth challenges, financial difficulties, and the necessity to balance everyday life. These results underscore the extensive burden on caregivers, necessitating targeted support and interventions to alleviate their multifaceted challenges (Table 2).

**Table 2:** CaTCoN questionnaire scores CaTCoN: Cancer Caregiving Consequences Inventory

CaTCoN questionnaire	Response (%)					Mean±SD
The extent to which the caretaker had to provide to the patient:	None	A little	Some	A lot	Do not know/not relevant	
Practical assistance	1	20	11	68	0	71.9±21.34
Personal care	49	26	12	8	0	67.8±24.5
Psychological support	3	20	32	42	0	78.8±23.4
	Not at all	To a low degree	To some degree	To a high degree	Do not know/not relevant	
Been partially responsible for tracking referrals and quick examinations and treatment correctly	50	23	20	7	0	24.35±12.34
Felt too much responsible for home care	0	12	8	80	0	21.34±11.36
Spent time in transportation of the patient for therapy	No, not at all	Yes, a little	Yes, some	Yes, a lot	Do not know/not relevant	
	0	5	6	87	0	57.89±24.51
Consequences of caregiving						
Cancer of the patient	No, not at all	Yes, a little	Yes, some	Yes, a lot	Do not know/not relevant	
Resulted in stress	2	10	15	73	0	63.4±12.34
Had a negative effect on your physical health	12	24	24	40	0	22.5±14.28
You need more time for other family members	2	2	54	36	6	54.23±34.56
You need more time for friends/acquaintances	2	2	54	38	4	53.45±32.16
Increased awareness of the essential things in life	4	22	40	30	0	26.4±24.3
Resulted in you making positive changes	42	10	12	36	0	37.56±22.34
Value for relationships has gone up	8	28	34	28	2	28.6±32.4
	Always or almost always	Mostly	Only sometimes	Rarely or never	Do not know or not relevant	
Afford to take time off, get leave from work, make similar arrangements	24	30	42	4	0	18.97±15.39
	Not at all	To a low degree	To some degree	To a high degree	Do not know/not relevant	
Your absence from work due to a patient’s illness has posed problems for you at the workplace	0	0	0	100	0	NA
You have borne adverse financial situations because of being a caregiver?	0	0	10	90	0	10.6±7.83
	No	Yes				
Did you ever need financial counseling	16	84				
Did you require a psychologist?	90	10				
	To a high degree	To some degree	To a low degree	Not at all	Do not know/not relevant	
Have you required to be able to take a break from the practical tasks?	60	24	10	6	0	31.24±18.97
Have you taken a break from tasks that are practical?	10	5	32	53	0	29.87±20.46
Felt the need to lead a "normal" life	60	24	10	6	0	31.24±18.97
Felt that you could lead a "normal" life	10	18	24	48	0	32.56±12.84

## Discussion

The technological advances made in the field of cancer therapeutics have helped to extend the lifespan of patients. Thus, prolonged survival means there is a need for continued tailored supportive care [9]. Caregivers of cancer patients manage medical tasks, emotional support, and daily activities, often juggling work and personal life. They face emotional strain, financial burdens, and physical exhaustion. Balancing their own needs with caregiving responsibilities can lead to burnout and stress, significantly impacting their mental and physical well-being. To support caregivers of cancer patients, implementing regular respite care can provide necessary breaks, while access to support groups and counseling can help manage emotional strain. Financial aid programs and flexible work arrangements can ease financial and time burdens. Additionally, offering training on medical tasks, promoting self-care, and building a network of community support services can significantly reduce physical exhaustion and stress, improving overall well-being. The caregiver population in this study predominantly comprises primary caregivers, with spouses/partners being the primary relationship to the patient [10,11]. Systematic review shows that spousal interventions positively affect cancer care and help improve the patient's and caregiver's quality of life [12]. A significant proportion of caregivers reported experiencing substantial caregiving workloads encompassing practical help, psychological support, and transportation. Negative consequences, such as stress, highlight the demanding nature of caregiving, potentially jeopardizing caregivers' well-being [13]. A three-year study by Moser et al. reported distress being prevalent even after a long time, though possibly the severity levels may have decreased among the spouses [14]. In the present study and across multiple studies, male or female spouses have been the primary studies. There is no recent literature to report the mental and emotional challenges a child undergoes when a parent is diagnosed with cancer. The young are presumably equally affected, and their stories must be highlighted in the burden of care. Despite no biased attempt, the sub-group of children as primary caregivers were few in the present study. Hence, we could not highlight the specific details related to this group to a large extent. Despite these challenges, positive experiences were also noted, emphasizing the complexity of the caregiver role. The multivariate regression analysis demonstrated a significant association between caregiver characteristics and various aspects of caregiving. Spouses/partners were significantly associated with specific questionnaire items (3, 4, 6, and 40), indicating a unique set of challenges and responsibilities experienced by spousal caregivers compared to those caring for children. Similar findings were also reported across several other recent studies from the West [15-18]. Notably, females and spouses/partners showed a strong correlation with increased workload, personal growth challenges, financial difficulties, and the need to maintain an everyday life. It could also be because our study population had more females and spouses as caregivers. However, this highlights the nuanced impact of caregiving responsibilities on different demographic groups within the caregiver population [19].

The analysis of the CaTCoN questionnaire scores further illuminates the caregiving experience. Noteworthy findings include the substantial responsibilities shouldered by caregivers, particularly in practical help, personal care, and psychological support domains. Additionally, caregivers expressed varying degrees of involvement in administrative tasks related to hospital referrals and managing home care responsibilities. The constant stress makes them wonder if they can ever live an everyday life. Specific individuals are reluctant to display joy, fearing their family members might misinterpret their happiness [20]. However, as found in the present study, these challenges bring positive outcomes, such as increased awareness and positive changes, predominantly among female spouses [21]. There is still a doubt if this compromises the situation or if they truly learn to fight back. Any caretaker's support under such circumstances should be addressed, even by themselves. While we explored only heterosexual couples in the present study, a significant gap exists in understanding what same-sex partners undergo. Their coping mechanisms are the same, especially in a more conservative society like India [22]. In the present study, we did not come across caregivers reporting needing more information from healthcare professionals. The review by Mbozi reported that the female caregivers were neglected, not communicated about the diagnosis, and overall showed little to no empathy [19]. Though such discrimination did not emerge in our findings, this still highlights the continued sensitizing of the healthcare personnel toward a holistic and inclusive approach toward cancer patients and their caregivers. Hence, the therapy should be more unit-specific, comprising cancer patients and caregivers, than patient-centered alone. This study provides a comprehensive understanding of caregiving responsibilities and the nuanced consequences of caregiving. It contributes valuable insights to the existing literature on caregiver experiences and can inform the development of targeted interventions and support programs.

The study has certain limitations as follows: 1. The study relies on snowball sampling, which can introduce bias and limit the generalizability of the results to a broader population. This sampling method may not capture the full diversity of caregivers for individuals with head, neck, and face cancer. 2. The sample may not be representative, potentially skewing overall conclusions. This limitation impacts the reliability of the findings, as the experiences of a broader, more varied caregiver population are not adequately reflected. 3. Caregivers may present their experiences in a way that conforms to social expectations, leading to social desirability bias. This can distort the accuracy of the data, affecting the validity of the study's conclusions.

## Conclusions

The study illuminated the diverse consequences of caregiving, showcasing the demanding nature of the role and the positive outcomes, such as increased awareness and positive changes, predominantly observed among female spouses. The analysis of the CaTCoN questionnaire scores provided valuable insights into caregivers' substantial responsibilities and varying degrees of involvement in caregiving domains. Despite specific challenges, positive experiences were noted, highlighting the complex nature of the caregiver role. The study underscored the importance of understanding caregivers' particular challenges with a call to explore the often-overlooked experiences of children as primary caregivers. In the broader context, this research contributes to the existing literature on caregiver experiences, offering valuable insights that can inform the development of targeted interventions and support programs. The study findings underscore the necessity for a holistic and inclusive approach to cancer care, urging healthcare professionals to be sensitized to the diverse needs of patients and their caregivers. Overall, this study enhances our understanding of the caregiving landscape in the challenging context of advanced cancer care.
